# Effect of Adult Male Diet on Mating Propensity and Competitiveness of the Asian Tiger Mosquito (*Aedes albopictus*)

**DOI:** 10.3390/insects17090902

**Published:** 2026-08-27

**Authors:** Stefania P. Kaltsou, Eleni C. Savvidou, Evaggelia D. Mpakovasili, Antonios Michaelakis, Nikos T. Papadopoulos

**Affiliations:** 1 Laboratory of Entomology and Agricultural Zoology, Department of Agriculture, Crop Production and Rural Environment, University of Thessaly, 38446 Volos, Greece; skaltsou@uth.gr (S.P.K.); eleni241986@gmail.com (E.C.S.); evamp789987@gmail.com (E.D.M.); 2 Laboratory of Insects & Parasites of Medical Importance, Scientific Directorate of Entomology and Agricultural Zoology, Benaki Phytopathological Institute, Kifissia, 14561 Athens, Greece; a.michaelakis@bpi.gr

**Keywords:** *Aedes albopictus*, Culicidae, male diet, mating success, competitiveness, Sterile Insect Technique

## Abstract

The Asian tiger mosquito, *Aedes albopictus*, is a major vector of arboviruses such as dengue, Zika and chikungunya, posing significant public health risks worldwide. Understanding male mating performance is crucial for optimizing control methods such as the Sterile Insect Technique (SIT). The aim of the current study was to investigate the influence of adult male diet on male *Ae. albopictus* mating success and competitiveness. Three adult diets were tested: 10% sugar solution, 10% molasses solution and 8% sugar solution enriched with 2% hydrolyzed yeast. Results revealed that males fed a molasses-based diet exhibited shorter latency time to mating and higher competitiveness against conspecifics. Male diet had no significant post-mating effects on females, neither due to fecundity nor to egg hatch rates. These findings underline the relevance of nutritional optimization in laboratory rearing. Although molasses-based diets appear promising for improving male quality, any implications for sterile-male release programs should be considered cautiously, as the present results derive solely from laboratory conditions.

## Simple Summary

Understanding how the diet of adult male mosquitoes influences their mating ability is critical for improving current, environmentally friendly control approaches such as the Sterile Insect Technique (SIT). The Sterile Insect Technique relies on releasing large numbers of mass-reared, sterilized males that can successfully compete with wild males to mate with feral females. In this study, we assessed how three adult diets affect the mating behavior and competitiveness of *Aedes albopictus*, an invasive mosquito species and an important vector of several human pathogens. The adult diets we used were: (a) 10% sugar solution, (b) 10% molasses solution and (c) an 8% sugar solution supplemented with 2% hydrolyzed yeast. Males fed with molasses showed consistently better mating performance. They initiated mating sooner, maintained longer mating durations and outcompeted males raised on a standard sugar diet. In contrast, males receiving the yeast-enriched diet did not exhibit improved performance and were less competitive than sugar-fed males. Despite these clear differences in male behavior, male diet did not influence female reproductive output. Females mated with males from all diet groups produced similar numbers of eggs and hatching rates remained unchanged.

## 1. Introduction

Mosquitoes (Diptera: Culicidae) are one of the largest groups of blood-feeding insects that cause nuisance and transmit numerous pathogens. Each year, mosquito-borne diseases infect millions of people, causing several hundred thousand deaths worldwide [1]. Recent global assessments highlight that incidences and geographic distribution of mosquito-borne diseases continue to increase, driven both by biological and environmental factors [2,3]. In recent decades, intensified global travel, international trade and climate change have facilitated mosquito range expansion. Consequently, many countries are experiencing the emergence of new infectious diseases, outbreaks of previously controlled ones and the re-emergence of endemic mosquito-borne pathogens, posing significant challenges to global public health and high economic impact [4,5].

*Aedes* (*Stegomyia*) *albopictus* (Skuse, 1895) is an aggressive mosquito species that not only vectors diseases but also causes high levels of nuisance, making everyday life difficult for people living in affected areas, hence harming tourism, leading to serious economic problems [5]. This invasive mosquito species (IMS) is of particular importance because it is a competent vector of several major infectious diseases, such as dengue, Zika, chikungunya, yellow fever and filariasis, and its management is challenging [6,7]. Originating from Southeast Asia and the islands of the Western Pacific and Indian Ocean, it has rapidly expanded its distribution to Africa, the Americas, Australia and Europe [8]. Recent modeling studies confirm that climate change will further increase habitat suitability for *Aedes* species across Europe in the coming decades [3,8,9,10]. Consequently, the global economic burden associated with *Aedes*-borne diseases is estimated to reach at least US$ 94 billion [5]. Although locally transmitted *Aedes*-borne diseases have not been reported in Greece, current climatic conditions and the extensive establishment of *Ae. albopictus* indicate a potential risk for future autochthonous transmission [11]. This concern is reinforced by the broader European situation, where *Ae. albopictus* has expanded rapidly since the 1990s and is now established not only in Mediterranean but in central European regions as well [12]. Its establishment has been directly linked with multiple autochthonous dengue and chikungunya outbreaks, including chikungunya events in Italy and France between 2007 and 2024 and recurrent dengue clusters reported in Croatia, France, Spain, and Italy from 2010 to 2024 [13].

Recent epidemiological analyses further indicate that once *Ae. albopictus* becomes established, the interval until the first local outbreak has shortened markedly, reflecting increasing environmental suitability and rising viral importation through travel [14]. Adding to these concerns is the recent detection of *Aedes aegypti* (L.) in Cyprus, where both *Ae. aegypti* and *Ae. albopictus* have now been recorded in coastal urban areas [15]. Together, these trends highlight the growing vulnerability of the Eastern Mediterranean to *Aedes*-borne diseases and underscore the need for strengthened surveillance and targeted vector control strategies [13]. In Greece, the Asian tiger mosquito, *Ae. albopictus*, the only IMS currently established in the country, was first detected in 2003–2004 in Corfu and Thesprotia (western Greece). Since then, it has spread rapidly across most regions, including Central Macedonia, Peloponnese, and Attica [12,15,16,17,18].

The reproductive biology of *Ae. albopictus* contributes substantially to its invasive success, as adults reach sexual maturity within two to three days post-emergence and can mate repeatedly throughout their lifespan. Mating typically occurs during short aerial encounters, and males exhibit a strongly polygynous strategy, maintaining high sperm reserves and transferring both sperm and seminal fluid proteins that influence female fertility [19]. However, repeated matings can deplete male sperm stores and reduce seminal fluid proteins, ultimately lowering mating success and the fertility of subsequently inseminated females. These factors are particularly relevant for sterile-male programs, where male quality, sperm availability, and mating competitiveness directly determine the effectiveness of field releases [20].

Male diet plays a decisive role in shaping reproductive performance in *Ae. albopictus*. Adequate nutritional intake enhances male vigor, increases energy reserves, and improves traits directly linked to mating success, such as courtship activity, insemination ability, and sperm production [21,22,23]. Nutritional stress during larval or adult stages can reduce body size, impair flight capacity, and decrease both sperm quantity and quality, ultimately lowering male fertility [24]. Because male nutritional condition directly influences the quality of sperm and seminal components transferred during mating, diet becomes a critical determinant of reproductive output and male competitiveness [25,26]. Overall, nutrition is a central factor in the reproductive biology of *Ae. albopictus*, affecting male mating success and shaping population performance. Sucrose solutions are widely used as the standard adult diet in mosquito research, but molasses represents a low-cost, readily available carbohydrate source with a rich sugar profile. Although it has not been previously evaluated as an adult diet, its accessibility and composition make it an interesting candidate for exploring alternative feeding regimes.

The SIT requires the continuous production and release of large numbers of sterile males that can effectively compete with wild males to mate with feral females. According to IAEA/WHO guidelines, successful SIT implementation depends on standardized mass-rearing procedures, strict quality-control monitoring, and the maintenance of key biological traits such as adequate body size, strong flight and dispersal ability, sufficient longevity, and robust mating performance. Ensuring that sterile males meet these quality criteria is essential for achieving high field competitiveness and maximizing the suppression efficiency of SIT programs [27,28].

Despite the importance of male nutritional status for mating competitiveness and female reproductive output, the specific effects of different adult male diets on key performance traits remain insufficiently understood. Addressing this gap is essential for improving laboratory rearing protocols and enhancing the efficiency of population-suppression approaches such as SIT. The aim of the present study was therefore to investigate the role of adult male nutrition on (a) male mating performance and (b) the life-history traits of female mates.

## 2. Materials and Methods

### 2.1. Insects Used and Laboratory Conditions

We used an *Ae. albopictus* population collected from Karitsa (39.844177, 22.761979), Larissa, Greece, in 2017 and kept since then under constant laboratory conditions in walk-in chambers (25 ± 1 °C, 65 ± 5% RH, and 14:10 (L:D) photophase, with lights turning off at 13:00). The laboratory colony has been maintained continuously under controlled conditions since its establishment, without the introduction of wild material. Mosquito rearing and maintenance were conducted under laboratory conditions following a previously published protocol [29], whereas egg hatching was performed according to a separate established protocol [30].

### 2.2. Effect of Male Diet on Mating Propensity

To assess the effect of male diet on mating propensity, pupae were collected daily from the rearing basins and placed individually in small Petri dishes (5 cm in diameter) containing 15 mL of water. Upon emergence adults were sexed, with males placed in 20 × 20 × 20 cm plexiglass cages at a density of 25 individuals per cage, while females were housed individually in smaller plexiglass cages (15 × 15 × 15 cm). Females had access to a 10% sugar solution as a food source. Diet solutions were prepared at the beginning of the experiment and remained available inside the cages throughout the adult maintenance period. Males were divided according to adult diet treatment and provided with one of the following:

(A) 10% sugar solution, (S 10%, control);
(B) 10% molasses (Fytro Melasa, Athens, Greece) solution, (M 10%);
(C) 8% sugar solution plus 2% hydrolyzed brewer’s yeast (S + HBY 10%).

Food was available through a plastic container with a small piece of Wettex^®^ wick cloth. Initially, 7-day-old females were given the opportunity to mate with 7-day-old males from the respective three diet treatments. The mating ratio was 1:1 (female: male). Food was removed from the observation cage before introducing the male. Mating behavior was observed from 12:00 to 14:00 (which reflects the 18:00–20:00 activity period under real field conditions). Both latency (time to initiate mating) and duration of mating were recorded. After the first mating occurred, the male was removed from female cages.

Subsequently, all mated females were offered a blood meal at the 9th day of age. For this, a volunteer placed her hand into each cage for 10 min and blood-feeding success was recorded. Blood meals were offered to the mosquitoes using the author’s forearm (S.P.K), with full informed consent, following medical treatment and the application of an appropriate anti-pruritic gel. On day 14, females that received a blood meal had access to an artificial oviposition substrate (moist filter paper in a small black plastic cup) for 3 days. Eggs were counted under a stereoscope, and embryonic development followed. Two weeks later, egg hatching was recorded following the previously described procedures.

Each replicate consisted of 60 males, including 20 fed with 10% sugar (S10), 20 fed with molasses (M10), and 20 fed with yeast (S + HBY), as well as 60 females maintained on a 10% sugar solution. With three replicates performed, the experiment involved a total of 180 males and 180 females. Each dietary treatment was evaluated on different days, with observations scheduled according to the specific age of the mosquitoes. This ensured that all measurements were performed at comparable physiological stages. To minimize observer-related variation, all assessments were conducted by the same investigators across all replicates and dietary treatments.

### 2.3. Effect of Male Diet on Mating Competitiveness

To assess the effect of male diet on mating competitiveness, the procedure followed for collecting pupae and separating adults by sex was the same as the one described above. Females had access to a 10% sugar solution, while males were separated by treatment and provided with one of the three previously described diets.

For the mating observation, two males from different diet treatments were placed in a single observation cage with one female. The pairwise combinations always included one male fed with the 10% sugar solution and one male fed with either the 10% molasses solution or the enriched sugar solution (S + HBY 10%).

One of the two males was marked with a special fluorescent powder to distinguish the treatment group and ensure visibility in the dark by using UV light. The coloring was alternated and equally distributed among treatments to avoid bias in female mate choice due to color marking.

Mating observations were conducted from 18:00 to 20:00 (which reflects the 18:00–20:00 activity period under real field conditions), and both mating latency and duration were recorded. The same procedure described above was followed for blood-feeding of mated females, oviposition, and egg hatching. Recorded data included blood-feeding success, number of eggs laid, and larval hatching success.

Three replicates were conducted for each diet comparison. Specifically, for the first diet comparison, 30 females fed on a 10% sugar solution, 30 males fed on a 10% sugar solution, and 30 males fed on a 10% molasses solution were used (in each replicate). With three replicates performed, the experiment involved a total of 180 males and 90 females Similarly, the same procedure was followed for the comparison of the diet 10% sugar solution with 8% sugar + 2% hydrolyzed brewer’s yeast solution.

### 2.4. Data Analysis

The effect of male diet on mating success was assessed using binary logistic regression, and pairwise comparisons were performed with the chi-square test. Effects of diet on mating duration and latency were evaluated with the Cox proportional hazards model, while pairwise comparisons between treatments were carried out using the log-rank test [31]. The effect of diet on blood-feeding was examined using binary logistic regression, and pairwise comparisons were performed with the chi-square test. For both fecundity and egg hatching rates we used one-way ANOVA. Normality was evaluated using Shapiro–Wilk tests and inspection of Q–Q plots, while homogeneity of variances was examined with Levene’s test. All assumptions were met, and therefore one-way ANOVA with Bonferroni post hoc comparisons was applied. The statistical software IBM SPSS Statistics for Windows, Version 26.0 (IBM, Armonk, NY, USA), was used for data analysis. In all tests above the significance level was set at α = 0.05.

## 3. Results

### 3.1. Effect of Male Diet on Mating Propensity

**Figure 1:**
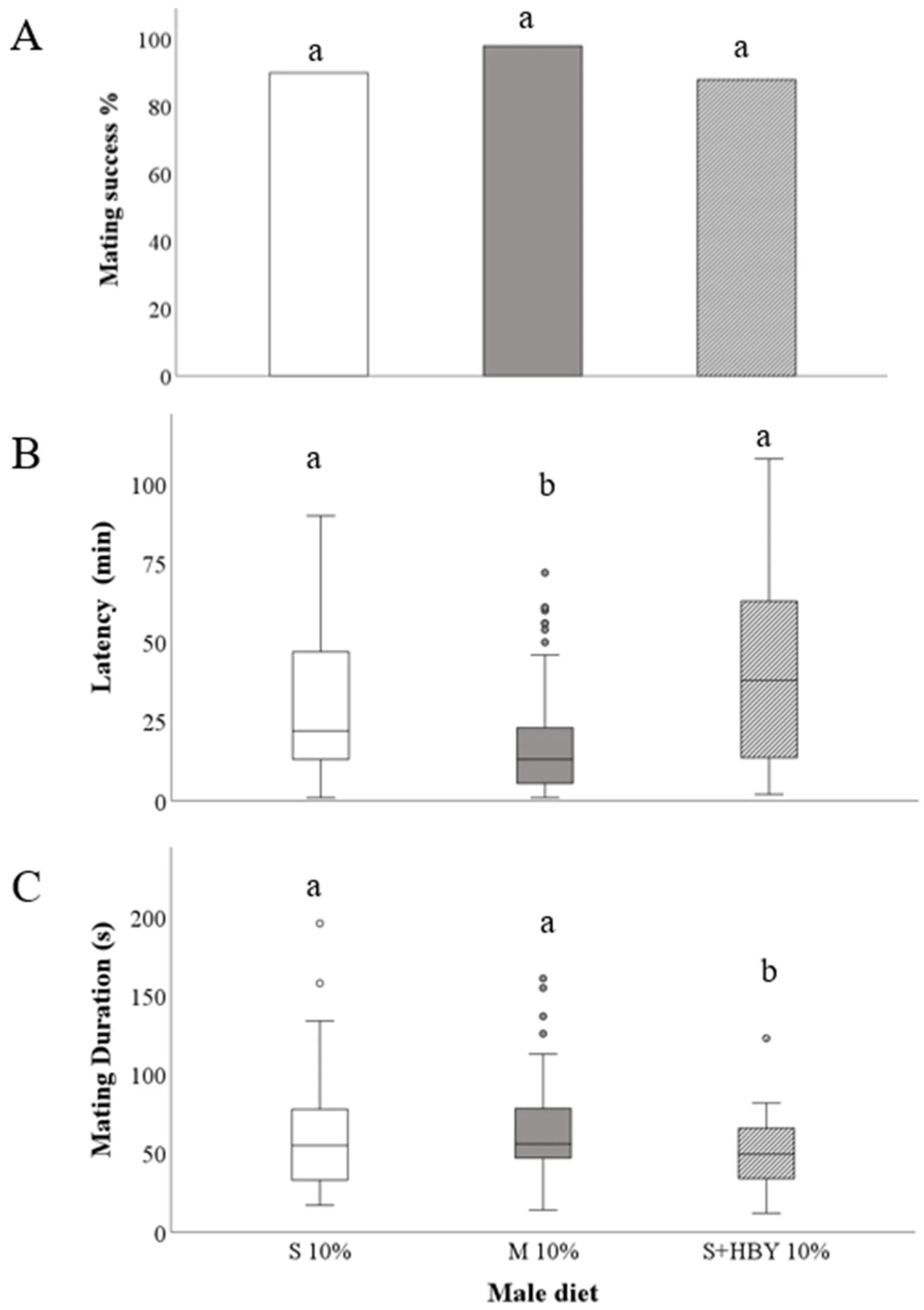
Mating success ((**A**), proportion), latency to mate time ((**B**), boxplots) and mating duration ((**C**), boxplots) of males that were fed different diets. Different letters above bars indicate significant differences (*p* < 0.05). Circles in boxplots denote outlier values falling outside the whisker range. S10% = 10% sugar solution; M 10% = 10% molasses solution; S + HBY 10% = 8% sugar solution enriched with 2% hydrolyzed brewer’s yeast.

Male diet did not affect mating success, as indicated by the chi-square test (*χ*^2^ = 4.933, df = 2, *p* = 0.085; Figure 1A). Because the overall test was not significant, no pairwise comparisons were performed. No significant differences were detected between S10% and S + HBY (*p* = 0.634). The Cox regression analysis showed that male diet significantly affected latency time (Wald test = 18.131, df = 2, *p* < 0.001; Figure 1B). Males fed M10% mated significantly sooner than those fed S10% or S + HBY 10% (log-rank test). Male diet also affected mating duration (Wald *χ*^2^ = 6.430, df = 2, *p* = 0.040). Males fed S10% and M10% engaged in longer matings compared to males fed S + HBY 10% (Figure 1C).

**Figure 2:**
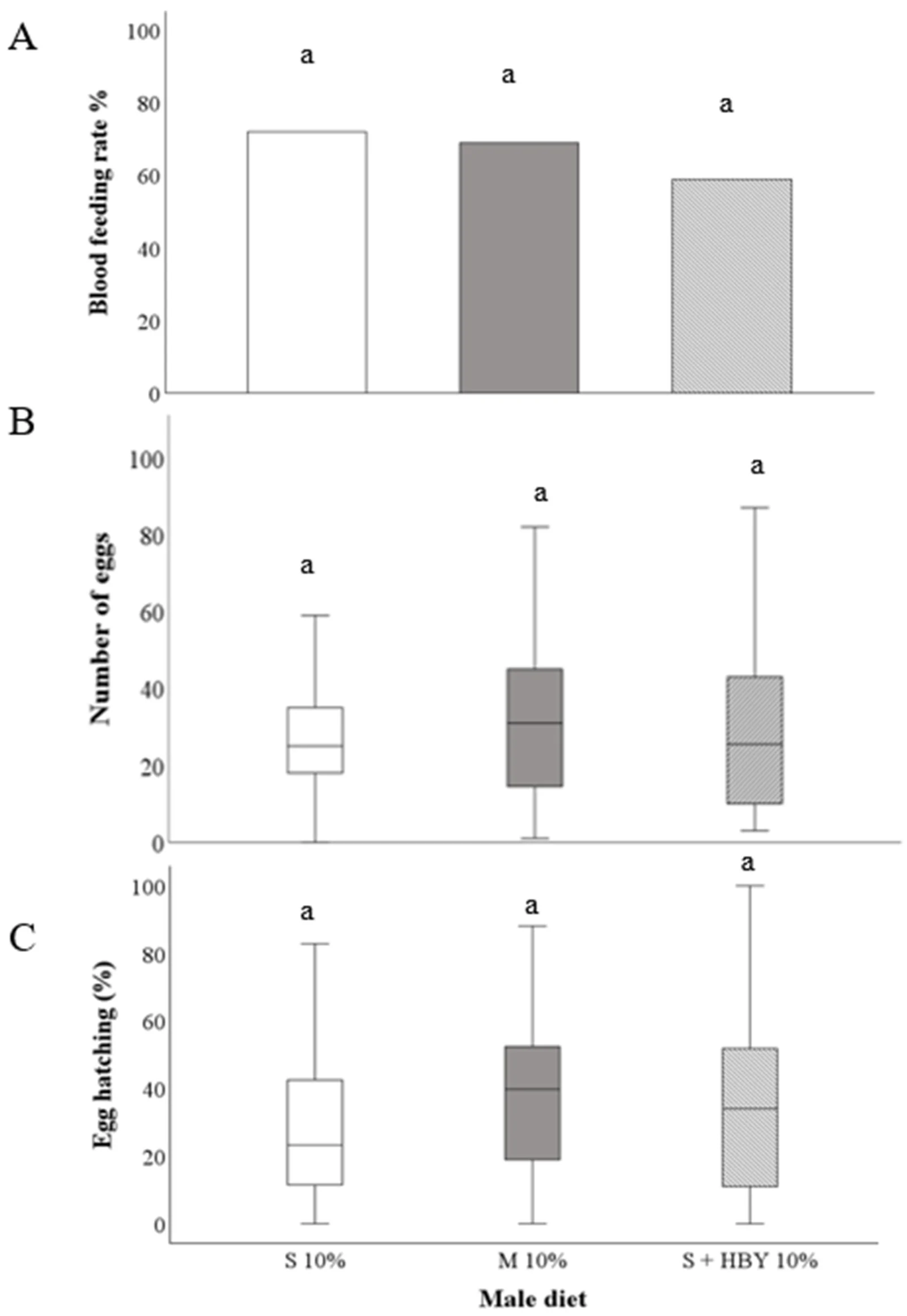
Blood-feeding success ((**A**), proportion), fecundity ((**B**), boxplots), and egg hatching rate ((**C**), boxplots) of females mated with males provided with different adult dietary treatments. Identical letters above bars indicate no significant differences (*p* > 0.05). S 10% = 10% sugar solution; M 10% = 10% molasses solution; S + HBY 10% = 8% sugar solution enriched with 2% hydrolyzed brewer’s yeast.

Male diet did not affect any of the reproductive parameters measured in females mated with them (*p* > 0.05). Blood-feeding success was similar among females mated with males fed S10%, M10% or S + HBY 10% (Wald *χ*^2^ = 2.622, df = 2, *p* = 0.270; Figure 2A). Likewise, male diet did not affect the egg production of female mates (*F*_2, 107_ = 0.553, df = 2107, *p* = 0.577; Figure 2B). Finally, egg hatching was similar among females mated with males fed S10%, M10% or S + HBY 10% (*F*_2, 106_ = 1.978, df = 2, *p* = 0.143; Figure 2C).

### 3.2. Effect of Male Diet on Mating Competitiveness

#### 3.2.1. Sugar (S10%)-Fed Compared to Molasses (M10%)-Fed Males

**Figure 3:**
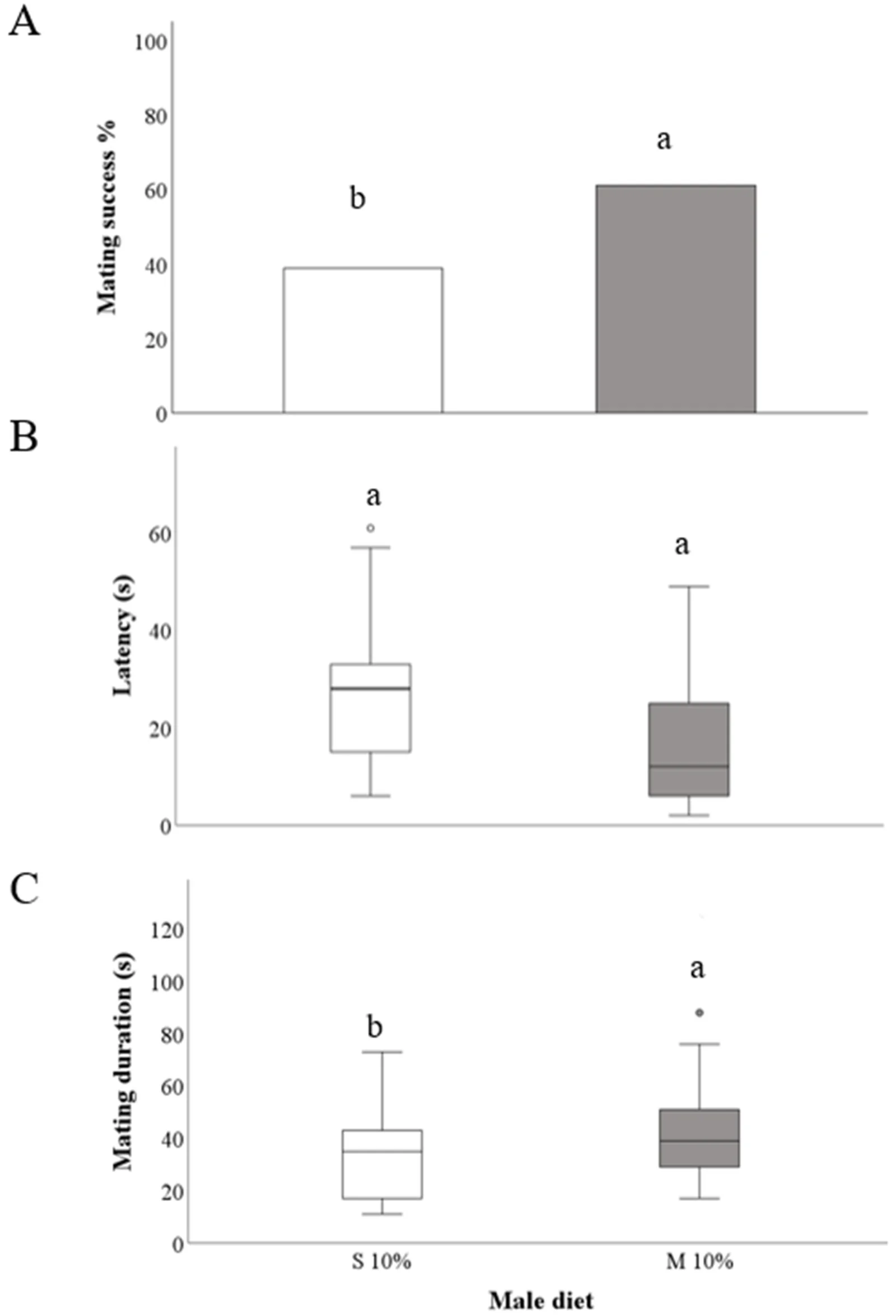
Mating success ((**A**), proportion), latency to mate time ((**B**), boxplots) and mating duration ((**C**), boxplot) of males fed either 10% sugar solution (S 10%) or 10% molasses solution (M 10%). Different letters above bars indicate significant differences (*p* < 0.05). Circles in boxplots denote outlier values falling outside the whisker range.

Male diet had a significant effect on mating competitiveness with mating success being higher for males fed M10% compared to those fed S10% (*χ*^2^ = 8.81, df = 1, *p* = 0.002; Figure 3A). In contrast, male diet did not influence shorter mating latency (Wald *χ*^2^ = 0.367, df = 1, *p* = 0.545; Figure 3B). However, mating duration was longer for males fed M10% compared to those fed S10% (Wald *χ*^2^ = 6.374, df = 1, *p* = 0.012; Figure 3C).

**Figure 4:**
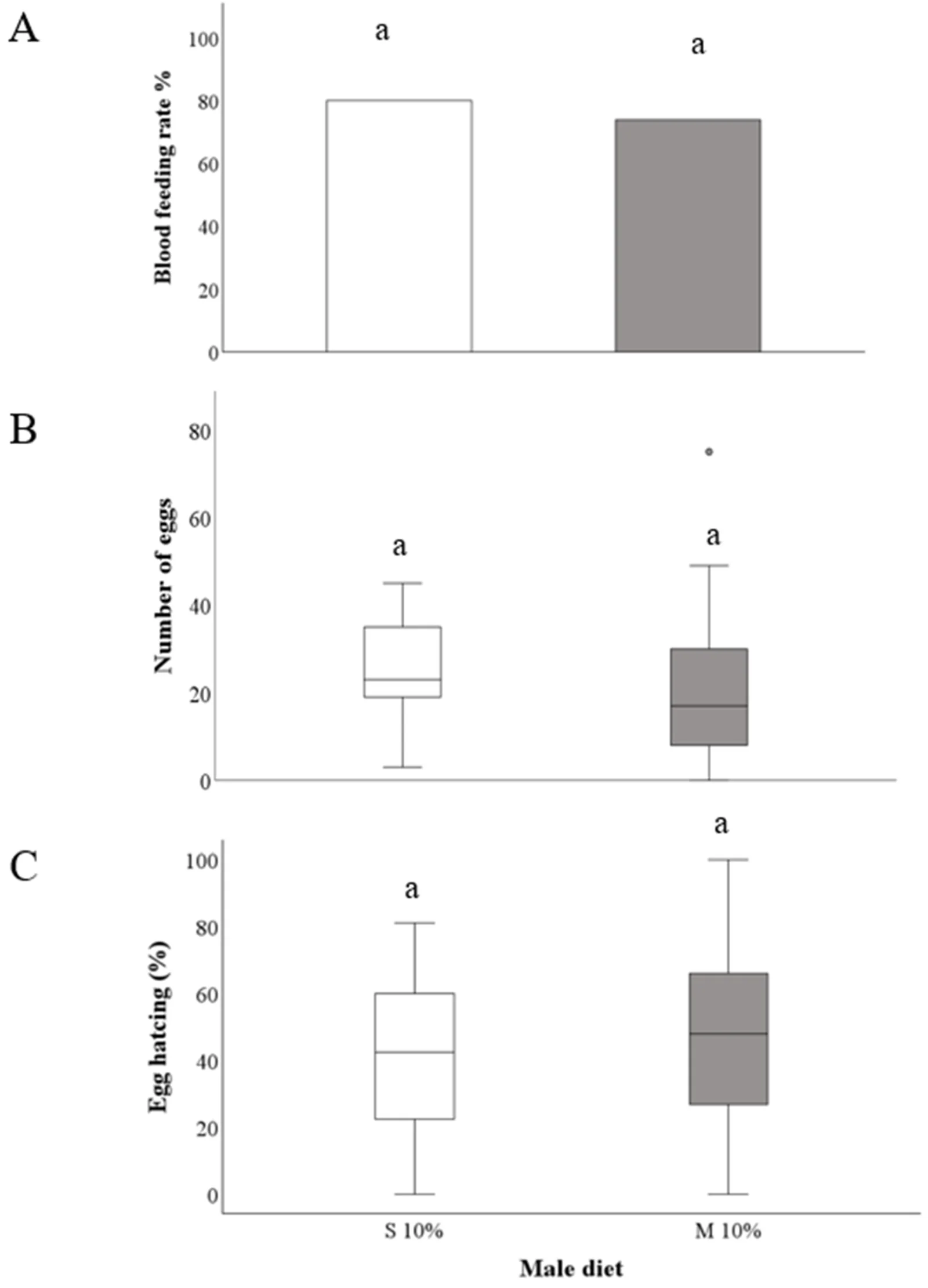
The effect of adult male food (10% sugar solution (S 10%), 10% molasses solution (M 10%)) on ((**A**), proportion) percentage of blood-feeding success, ((**B**), boxplots) the number of eggs laid, and ((**C**), boxplots) egg hatching rates of females after mating with males fed different food. Identical letters above bars indicate no significant differences (*p* > 0.05). Circles in boxplots denote in boxplots outlier values falling outside the whisker range.

Male diet had no effect on female blood-feeding rate. Blood-feeding rate of females mated with either S10% or M10% males was approximately 75% (Wald *χ*^2^ = 0.302, df = 1, *p* = 0.583; Figure 4A). Likewise, egg production was similar between females mated with males from the two dietary groups (one-way ANOVA: *F*_1, 65_ = 0.060, *p* = 0.809; Figure 4B). Egg hatch rates of females mated with the above male categories was also similar (*F*_1, 63_ = 0.501, *p* = 0.481; Figure 4C).

#### 3.2.2. Sugar (S10%)-Fed Compared to Sugar and Yeast (S + HBY10%)-Fed Males

**Figure 5:**
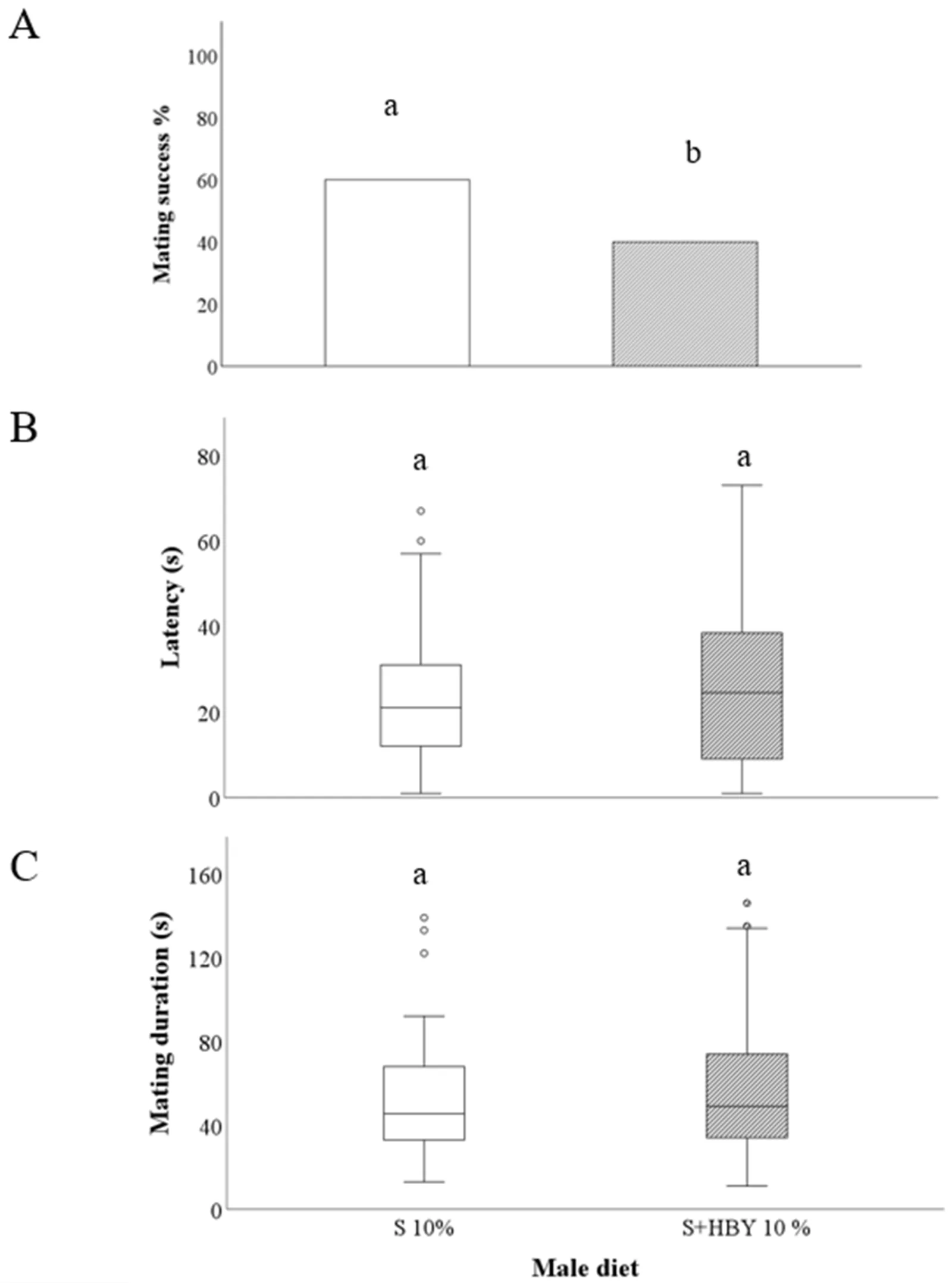
Mating success ((**A**), proportion), latency to mate time ((**B**), boxplots) and mating duration ((**C**), boxplots) of males that had access to either 10% sugar solution (S 10%) or 8% sugar solution enriched with 2% hydrolyzed brewer’s yeast (S + HBY 10%). Different letters above bars indicated significant differences (*p* < 0.05). Circles in boxplots denote outlier values falling outside the whisker range.

Overall mating competitiveness was higher for males fed S10% compared to those fed S + HBY 10%. Pairwise comparisons demonstrated that males fed S10% had higher mating competitiveness compared to those fed S + HBY 10% (*χ*^2^ = 7.22, d = 1, *p* = 0.007; Figure 5A). In contrast, male diet did not influence latency time (Wald *χ*^2^ = 1.208, df = 1, *p* = 0.272; Figure 5B). Similarly, mating duration was similar between the two male categories (Wald *χ*^2^ = 0.055, df = 1, *p* = 0.814; Figure 5C).

**Figure 6:**
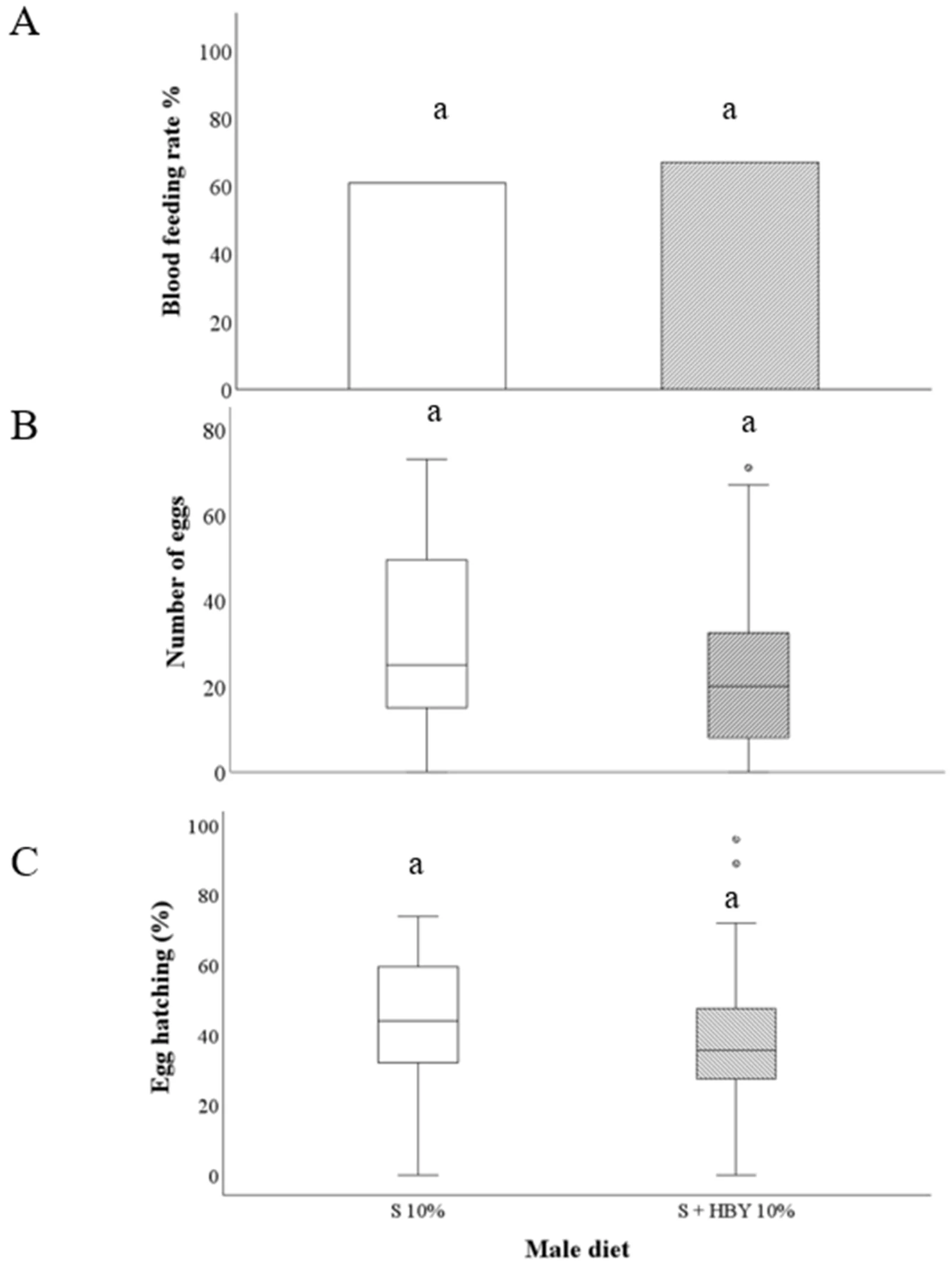
The effect of adult male food (10% sugar solution (S 10%) or 8% sugar solution enriched with 2% hydrolyzed brewer’s yeast (S + HBY 10%)) on percentage of blood-feeding success of females ((**A**), proportion), the number of eggs laid by females ((**B**), boxplots), and the hatching of eggs that came from females ((**C**), boxplots) that mated with the corresponding males. Different letters above bars indicate no significant differences (*p* < 0.05). Circles in boxplots denote outlier values falling outside the whisker range.

The diet of males (fed either S10% or S + HBY 10%) did not affect any of the reproductive parameters measured in females, including blood-feeding rates (Wald *χ*^2^ = 0.075, df = 1, *p* = 0.784; Figure 6A), the number of eggs oviposited by females mated with males from the two dietary treatments (*F*_1, 54_ = 1.240, *p* = 0.270; Figure 6B), and the hatch rates of the produced eggs (*F*_1, 53_ = 0.449, *p* = 0.506; Figure 6C).

## 4. Discussion

Our results revealed that males fed a molasses (M10%) diet exhibited higher mating success and better mating characteristics compared to males fed on the other adult diets (S10% and S + HBY 10%). In contrast, female parameters such as blood-feeding success, egg production, and larval hatching rates were not affected by the adult diet of their male mating partner. Male mating competitiveness was higher for males fed M10% compared to S10%. On the other hand, comparison between males fed S + HBY 10% and S10% showed a higher female mating preference for males fed on the S10% diet.

Adult mosquito diet has been recognized as a critical factor influencing reproductive success [32]. Males feed exclusively on sugar sources such as nectar and honeydew, which provide the energy required to survive and perform swarming and mating [33]. Previous studies have demonstrated that food quality can affect not only lifespan but also mating traits, including latency to mating and male competitiveness [34]. Additionally, different sugar sources have been shown to alter female reproductive physiology, with females fed on nectar inducing higher vitellogenin gene expression and increased egg production compared to those fed on honeydew [33]. Similarly, larval diet has been reported to shape adult quality, endurance, and mating competitiveness, which is particularly important for the effectiveness of the SIT. Studies over the last decade have shown that carbohydrate quality and availability strongly influence male energetic reserves, flight capacity, and courtship vigor, all of which directly affect mating competitiveness in the field [22,25]. Moreover, nutritional enhancement has been linked to improved male survival and sustained mating activity under semi-field conditions, suggesting that optimized adult diets can significantly increase the operational efficiency of sterile-male releases [35,36]. Our results align with these observations, demonstrating that molasses-fed males exhibit superior mating traits compared to those fed standard sugar solutions or yeast-enriched diets. This indicates that not only the quantity but also the type of carbohydrate source can modulate male reproductive performance. Although molasses-fed males showed improved performance, the underlying mechanism remains unclear. A plausible explanation is that molasses could enhance energetic condition or aspects of reproductive physiology; however, in the absence of direct measurements of energy reserves or sperm quality, this hypothesis remains speculative and should be interpreted with caution. Importantly, the absence of diet-related effects on female fecundity and egg viability suggests that nutritional benefits act primarily through male behavioral and physiological enhancement rather than through post-mating modulation of female reproductive output. An additional possibility is that the improved mating performance of molasses-fed males may reflect differences in overall energy availability rather than a specific nutritional effect of molasses. This explanation cannot be confirmed without direct measurements of energetic reserves, but it is consistent with the observed patterns. Taken together, these findings reinforce the importance of integrating adult nutritional optimization into mass-rearing protocols, as improving male mating competitiveness is a critical factor for maximizing the success of SIT-based suppression strategies in *Ae. albopictus* populations. Similar patterns have been reported for *Aedes aegypti*, where adult carbohydrate availability and sugar type have been shown to influence male mating success, energy reserves, and overall reproductive performance [26]. These parallels between species reinforce that adult nutritional quality is a key determinant of male fitness across *Aedes* mosquitoes. Our findings extend this knowledge by showing that adult nutrition can exert an equally important effect. From an operational perspective, the use of molasses could offer practical advantages for mass-rearing facilities, as it is inexpensive, widely available, and easy to handle at large scale [37]. These features may reduce production costs compared to refined sugars. However, potential limitations, such as variability in composition among commercial sources or the need for quality control, should also be considered when evaluating its suitability for SIT programs. The urgent need to develop tools for mosquito control (e.g., SIT approaches) has led to increased interest in the nutrition of adult mosquitoes, which can enhance specific traits in the adults. The SIT requires the production of males with enhanced fitness, capable of competing successfully with wild males [38]. Adult male longevity was not evaluated in the present study, even though it represents an important trait for SIT performance; its omission should therefore be acknowledged as a limitation. Nevertheless, ongoing experiments in our laboratory indicate that adult diet can also modulate male lifespan, with preliminary observations suggesting diet-dependent differences in survival. These early findings are consistent with the patterns reported here for male mating performance, further supporting the notion that adult nutritional quality influences multiple fitness-related traits in *Ae. albopictus*.

One of the findings from mating competitiveness experiments was that molasses as an adult male diet resulted in superior mating traits (shorter time to mating and longer duration) compared to the S10% solution. The superior mating performance of molasses-fed males may be partially explained by the nutritional composition of molasses, which contains small amounts of protein, amino acids, minerals (e.g., potassium, magnesium, iron), and micronutrients that are absent from simple sugar solutions. Micronutrients and amino acids are known to enhance reproductive physiology, flight performance, and overall male fitness in insects [39,40,41]. Such components may contribute to improved energetic reserves and sustained courtship activity, ultimately increasing male mating competitiveness.

In contrast, the yeast-enriched sugar solution did not improve male mating performance against the S10% solution. Although protein supplementation has been shown to enhance male competitiveness in other insects, particularly in Tephritidae [39,41], this effect is not consistently observed in mosquitoes. Male mosquitoes rely almost exclusively on carbohydrates for energy, flight performance, and mating activity, and do not efficiently utilize dietary protein [42]. On the other hand, the digestion of the yeast hydrolysate included in the offered diet may differ between male mosquitoes and male fruit flies. These may explain why the yeast-enriched sugar solution did not improve mating competitiveness in our experiments.

Despite the influence of male diet on mating behavior, female reproductive traits remained unaffected. Female fecundity and egg viability are primarily determined by the female’s own energetic reserves and blood meal quality, rather than by the nutritional status of the male [42]. Although seminal fluid proteins transferred during mating can modulate certain reproductive genes [33], these effects do not typically change egg number or hatching success in *Aedes* species. Our results align with this broader pattern, indicating that while male diet enhances mating competitiveness, it does not exert downstream effects on female productivity.

Mating competitiveness in mosquitoes is a crucial factor in controlling vector populations through strategies such as SIT [38]. Studies have investigated various parameters influencing mosquito mating, including sterilization methods, field competitiveness, dose–response relationships, and genetic factors. Adult male diet can confer desirable traits to mosquitoes, making them more competitive against wild males [43,44].

Although the present results indicate that molasses may support traits relevant to SIT efficiency, this inference should be made with caution, as performance under controlled laboratory conditions does not necessarily translate into field competitiveness. Further research, including field-based evaluations, is therefore required to substantiate these findings. Finally, it should also be acknowledged that the study was based on a single mosquito population and evaluated only three adult diets, which places the conclusions within a specific experimental context.

## 5. Conclusions

Overall, our study contributes new evidence to the existing literature by identifying molasses as a particularly effective diet for laboratory-reared *Ae. albopictus* males. Its use can improve the production of males with enhanced competitiveness, which has direct practical implications for SIT applications and the suppression of wild populations of this species. Limitations of the current study include the restricted number of dietary treatments and the fact that mating competitiveness was assessed under controlled laboratory conditions, which may not fully reflect field performance. Additionally, the study focused solely on adult male diet without evaluating potential carry-over effects from larval nutrition. Future work incorporating semi-field trials, multiple diet regimes, and long-term fitness assessments would provide a more comprehensive understanding of how diet influences male reproductive success. Although molasses appears to be a promising dietary component for enhancing traits relevant to SIT efficiency, its practical use in operational programs should be considered preliminary, as further validation through semi-field and field experiments is required to substantiate these indications. Future research should prioritize validating the observed dietary effects under semi-field and field conditions and exploring how diet interacts with other key biological parameters, such as longevity and mating performance, to optimize SIT outcomes.

## Data Availability

The data presented in this study are available upon request from the corresponding author.

## Abbreviations

The following abbreviations are used in this manuscript:

IMS	Invasive mosquito species
SIT	Sterile Insect Technique
S 10%	10% sugar solution
M 10%	10% molasses solution
S + HBY 10%	8% sugar solution plus 2% hydrolyzed brewer’s yeast
